# Characterisation and Phylogenetic Analysis of the Complete Plastid Genome of *Physalis minima* L. from Malaysia

**DOI:** 10.21315/tlsr2025.36.3.14

**Published:** 2025-10-31

**Authors:** Wei Lun Ng, Rebecca Jiayiin Ng, Douglas Law, Qiuying Ren, Charles Gnanaraj, Yoong Soon Yong, Shiou Yih Lee

**Affiliations:** 1 China-ASEAN College of Marine Sciences, Xiamen University Malaysia, Jalan Sunsuria, Bandar Sunsuria, 43900 Sepang, Selangor, Malaysia; 2 Faculty of Health and Life Sciences, INTI International University, Persiaran Perdana BBN Putra Nilai, 71800 Nilai, Negeri Sembilan, Malaysia; 3 Faculty of Pharmacy and Health Sciences, Royal College of Medicine Perak, Universiti Kuala Lumpur, No 3, Jalan Greentown, 30450 Ipoh, Perak, Malaysia; 4 R&D Quality Department, Osmosis Nutrition Sdn. Bhd., Lot 16014, Jalan Nilam 3, Bandar Nilai Utama, 71800 Nilai, Negeri Sembilan, Malaysia; 5 Faculty of Liberal Arts, Shinawatra University, Bang Toei, Sam Khok District, 12160 Pathum Thani, Thailand; 6 College of Ocean and Earth Sciences, Xiamen University, Xiang’An South Road, Xiang’An, 361102 Xiamen, Fujian, China

**Keywords:** Genetic Resources, Next-Generation Sequencing, Physaleae, Chloroplast Genome, Solanaceae, Sumber Genetik, Penjujukan Generasi Akan Datang, Physaleae, Genom Kloroplas, Solanaceae

## Abstract

*Physalis minima* L. is an herbaceous plant with ethnobotanic importance across many Asian cultures. In this study, we sequenced and assembled the plastid genome (plastome) of a P. minima sample from Malaysia, and conducted intraspecific pairwise and phylogenetic analyses with available data of its relatives. Our sample had a plastome of 156,973 bp in size with a GC content of 37.5%. The genome was circular, consisting of a large single-copy of 87,196 bp, a small single-copy of 18,447 bp and a pair of inverted repeats of 25,665 bp each. A total of 129 genes were annotated, including 84 CDSs, 37 tRNAs and eight rRNAs. Between the China and Malaysia accession, 458 variable sites were identified, and the pairwise distance was 0.003. Phylogenetic analysis was conducted using the complete plastome sequence based on the maximum likelihood and Bayesian inference methods. The findings revealed that our sample was significantly differentiated from the accession from China, and that both *P. minima* accessions clustered away from *P. angulata*, the synonym suggested by certain taxonomic authorities. This study facilitates precise taxonomic identification of P. minima within ethnomedicinal frameworks, enabling its distinction from closely related or putatively synonymous species that may exhibit divergent phytochemical compositions. These results provide important insights into the genetic diversity and taxonomic status of P. minima, and support its informed use in future research, conservation, and medicinal applications.

## Abstract (fr)

**Abstrak:**

Physalis minima L. ialah tumbuhan herba yang mempunyai kepentingan etnobotani dalam banyak budaya Asia. Dalam kajian ini, kami menjujuk dan menyusun genom plastid (plastom) bagi satu sampel P. minima dari Malaysia, serta menjalankan analisis pasangan dan filogenetik intraspesifik menggunakan data yang tersedia daripada spesies yang berkaitan dengannya. Sampel kami mempunyai plastom bersaiz 156,973 bp dengan kandungan GC sebanyak 37.5%. Genom ini berbentuk bulat, terdiri daripada satu salinan besar tunggal (87,196 bp), satu salinan kecil tunggal (18,447 bp) dan sepasang ulangan terbalik (25,665 bp setiap satu). Sebanyak 129 gen berjaya dianotasi, termasuk 84 CDS, 37 tRNA, dan lapan rRNA. Antara aksesi China dan Malaysia, 458 tapak berubah dikenal pasti, dengan jarak pasangan sebanyak 0.003. Analisis filogenetik dijalankan menggunakan jujukan lengkap plastom berdasarkan kaedah kemungkinan maksimum dan inferens Bayesian. Dapatan kajian menunjukkan bahawa sampel kami berbeza dengan ketara daripada aksesi China, dan kedua-dua aksesi P. minima mengelompok jauh daripada P. angulata, sinonim yang dicadangkan oleh pihak berkuasa taksonomi tertentu. Kajian ini memudahkan pengenalpastian taksonomi yang tepat bagi P. minima dalam kerangka etnomedik, membolehkan pembezaannya daripada spesies yang berkait rapat atau dianggap sinonim yang mungkin mempunyai komposisi fitokimia berbeza. Hasil kajian ini memberikan pandangan penting mengenai kepelbagaian genetik dan status taksonomi P. minima, serta menyokong penggunaannya secara berinformasi dalam penyelidikan, pemuliharaan, dan aplikasi perubatan pada masa akan datang.

**HIGHLIGHTS**

- The complete plastid genome of a *Physalis minima* sample from Malaysia was sequenced, assembled and annotated.
- The *P. minima* plastid genome was circular and 156,973 bp in size, and had a GC content of 37.5% and 129 annotated genes.
- Phylogeny showed genetic differentiation between accessions from Malaysia and China, and both clustered away from *P. angulata*.

## INTRODUCTION

The *Physalis* (Solanaceae) plant genus, which includes around 120 species, is primarily found in the Americas, Europe and Asia (Liang *et al*. 2024). In Malaysia, *P. minima* L. 1753 is the only native *Physalis* species recorded in the country (MyBIS 2024). The plant is traditionally consumed for treating illnesses such as fever, constipation, sore throat, stomachache, gastric and diabetes. (Lem *et al*. 2022). Research has shown that it is a phytochemically rich medicinal plant containing flavonoids, alkaloids and antioxidants. It exhibits notable antidiabetic, anti-inflammatory, analgesic, antimicrobial and diuretic activities (Novita *et al*. 2020). Due to its bioactive profile and edible, nutrient-dense fruit, the plant presents significant potential for application in pharmaceutical development, functional food products and the broader nutraceutical industry. However, the plastid genome (plastome) sequences of only 13 *Physalis* species are publicly available. For *P. minima*, a record for the complete plastome sequence of an accession from Shandong, China, was published (GenBank accession no. MH045577; Feng *et al*. 2020). Plastomes are essential for elucidating plant evolutionary relationships and taxonomy, given their conserved structure and phylogenetically informative mutation rates. They enable the resolution of complex lineages, identification of hypervariable regions for species delimitation, and detection of cryptic diversity. Such insights are vital for refining classifications and informing conservation strategies, particularly in taxa with threatened or morphologically similar species (Xu *et al*. 2022). As an important herb in Malaysia, the genomic information of *P. minima* is currently lacking. In this study, we sequenced and assembled the plastome sequence of P. minima from Malaysia using next-generation sequencing (NGS) technology and bioinformatics tools. As Shandong, China, has a profoundly different climate compared to Malaysia, we assessed the intraspecific pairwise analysis and genomic variation of *P. minima* between the two accessions and performed phylogenetic analysis to determine the molecular placement of *P. minima* from Malaysia among other *Physalis* taxa.

## MATERIAL AND METHODS

**FIGURE 1:**
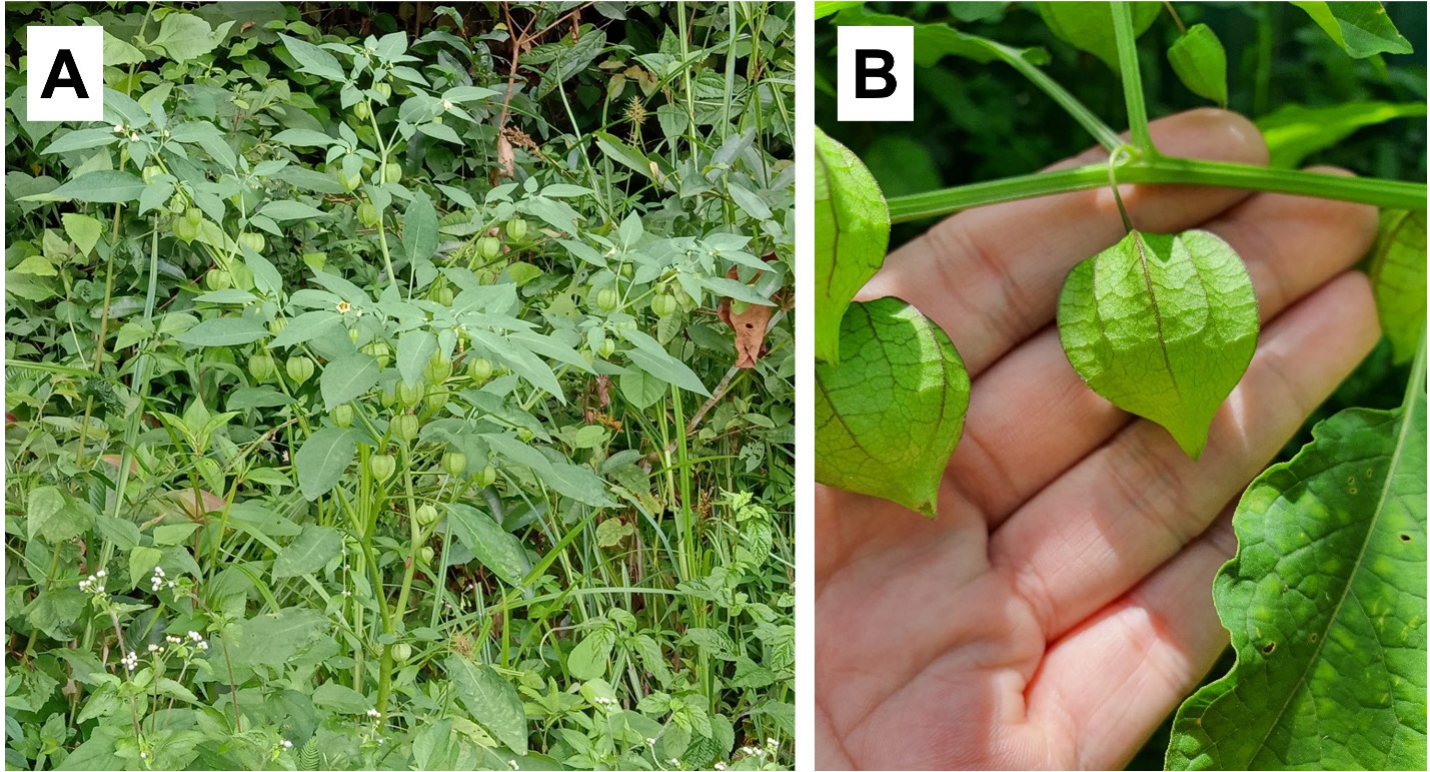
*Physalis minima* (A) whole plant in its habitat, (B) fruits (Photos by Y.S. Yong and C. Gnanaraj).

Fresh leaves of a *P. minima* individual (Fig. 1) were collected from a natural population in Ipoh, Perak, Malaysia (4.617991° N, 101.044373° E) and transported to the Biotechnology Laboratory of the Faculty of Health and Life Sciences, INTI International University, in a zip-lock bag containing silica gel. A voucher specimen with the collection number XMUM-PL-2023-YYS001 has been deposited at the Xiamen University Malaysia Specimen Collection (contact: Dr. Wei Lun Ng, weilun.ng@xmu.edu.my). Total genomic DNA extraction was performed using the DNeasy Plant Mini Kit (Qiagen, USA) following the manufacturer’s protocol. The DNA extract was quantified using a Qubit 4 fluorometer (Thermo Fisher Scientific, USA) for its purity and concentration prior to being sent to Guangzhou Jierui Biotechnology Company, Ltd. (Guangzhou, China) for next-generation sequencing (NGS).

A 350-bp paired-end genomic library was prepared using the TrueSeq DNA Sample Prep Kit (Illumina, USA), and 150-bp paired-end reads were produced on a NovaSeq 6000 platform (Illumina, USA). Approximately 3 Gb of raw NGS data was generated and fed into the NOVOwrap v1.20 (Wu *et al*. 2021) pipeline, based on the seed-and-extend algorithm, for the plastome sequence assembly process. The partial rbcL gene sequence of *Physalis minima* (GenBank accession no. MH767730) was selected as the seed sequence. By obtaining a single contig at the end of the plastome assembly step, gene annotation and identification of the IR regions were carried out using GeSeq v2.03 (Tillich *et al*. 2017), based on the BLAT-based homology search and the Chloë module. The annotated plastome sequence was manually checked for errors and the physical map was visualised using OGDraw v1.3.1 (Greiner *et al*. 2019).

To detect intraspecific genomic variation at the plastome level between the *P. minima* accessions from China and Malaysia, the two complete plastome sequences were aligned using MAFFT v7 (Katoh & Standley 2013). The number of variable sites was estimated using DnaSP v5 (Librado & Rozas 2009), and the intraspecific pairwise distance between the two accessions was calculated using MEGA7 (Kumar *et al*. 2016) based on the Kimura two-parameter (K2P) model under uniform rates and 1,000 bootstrap replicates. Gaps and missing data were treated as pairwise deletions. The model was selected for its capacity to differentiate substitution types, enhancing accuracy in closely related plastome comparisons.

To elucidate the phylogenetic relationship between *P. minima* from Malaysia and other related taxa, the complete plastome sequences of 18 other Physaleae accessions were included in a phylogenetic analysis: *Alkekengi officiniarum* var. *franchetii* (GenBank accession number: MH045575; Feng *et al*. 2020), *Iochoroma salpoanum* (GenBank accession number: KU315119; unpublished), *I. stenanthum* (GenBank accession number: KP262399; unpublished), *Physalis angulata* (GenBank accession number: MH045574; Feng *et al*. 2020), *P. angulata* var. *villosa* (GenBank accession number: OM257167; Zhan *et al*. 2022), *P. chenopodiifolia* (GenBank accession number: MN508249; Zamora-Tavares *et al*. 2020), *P. cordata* (GenBank accession number: MH045575; Sandoval-Padilla *et al*. 2020a), *P. ixocarpa* (GenBank accession number: OP748223; Zhang *et al*. 2022), *P. longifolia* var. *subglabrata* (GenBank accession number: OP748222; Zhang *et al*. 2022), *P. minima* (GenBank accession number: MH045577; Feng *et al*. 2020), *P. peruviana* (GenBank accession number: KP295964 and OP028208; unpublished), *P. philadelphica* (GenBank accession number: MN192191, MT254545, and MZ539568; Sandoval-Padilla *et al*. 2019; 2022b), *P. pubescens* (GenBank accession number: MH045576; Feng *et al*. 2020), *Whithania adpressa* (GenBank accession number: BK010847; Mehmood *et al*. 2020), *W. riebeckii* (GenBank accession number: BK010849; Mehmood *et al*. 2020). Two closely related species, *Capsicum annuum* (GenBank accession no. KR078313; Raveendar *et al*. 2015) and *C. baccatum* (GenBank accession no. KR078314; Kim *et al*. 2016) were also included as outgroups. The analysis was conducted using maximum likelihood (ML) and Bayesian inference (BI) methods through RAxML-HPC2 v8.2.12 (Stamatakis 2014) and MrBayes v3.2.7a (Ronquist *et al*. 2012), respectively, accessible via the CIPRES Science Gateway (Miller *et al*. 2011). For the ML tree, the general time reversible nucleotide substitution model (GTR) with gamma rate (+G) (=GTR+G) was selected, with branch support calculated under 1,000 bootstrap replicates. For the BI tree, a mixed number of substitution types and a 4-by-4 nucleotide substitution model were applied. Each Markov chain Monte Carlo was run for 2,000,000 generations, sampling at every 100 generations, with the first 25% of the trees discarded as burn-in. The final tree was visualised using FigTree version 1.4.4 (Rambaut 2018).

## RESULTS

**FIGURE 2:**
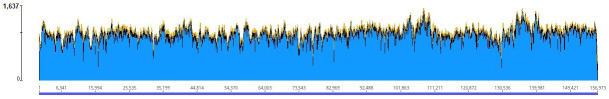
Read mapping depth (blue region) of the plastome sequence generated in this study. Y-axis shows the read mapping depth, while X-axis indicates the nucleotide position of the plastome.

**FIGURE 3:**
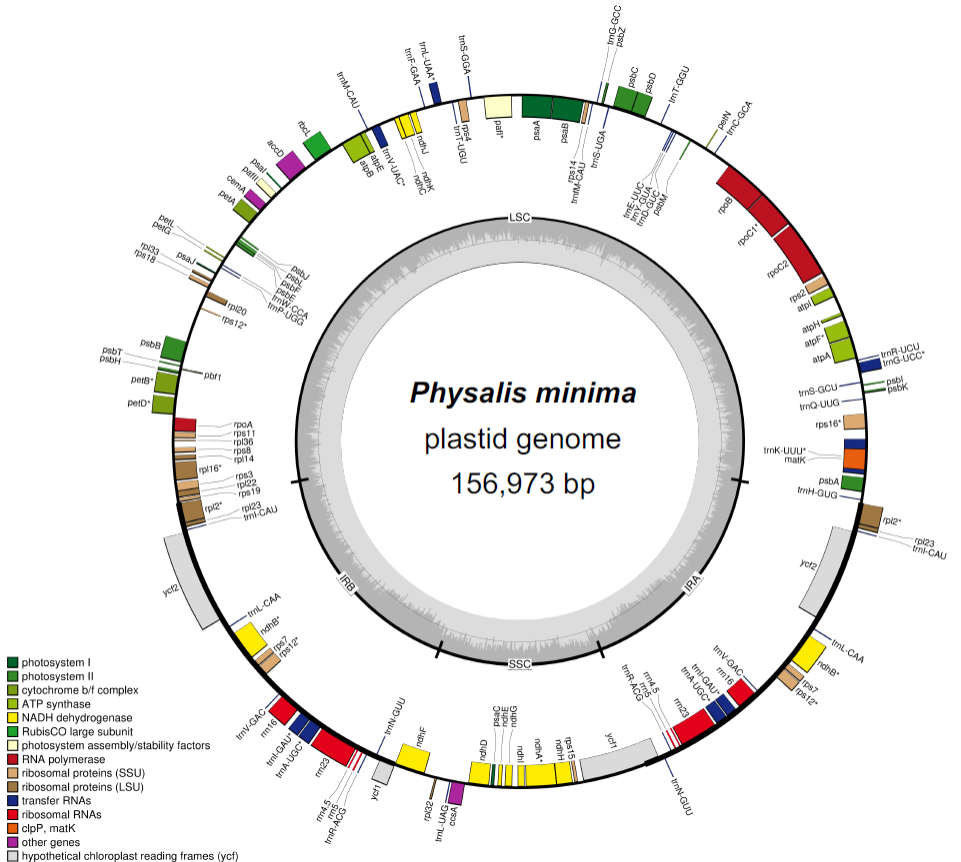
Plastid genome map of *Physalis minima*. Genes annotated in the circle are transcribed counterclockwise, while the genes annotated outside the circle are transcribed clockwise.

**FIGURE 4:**
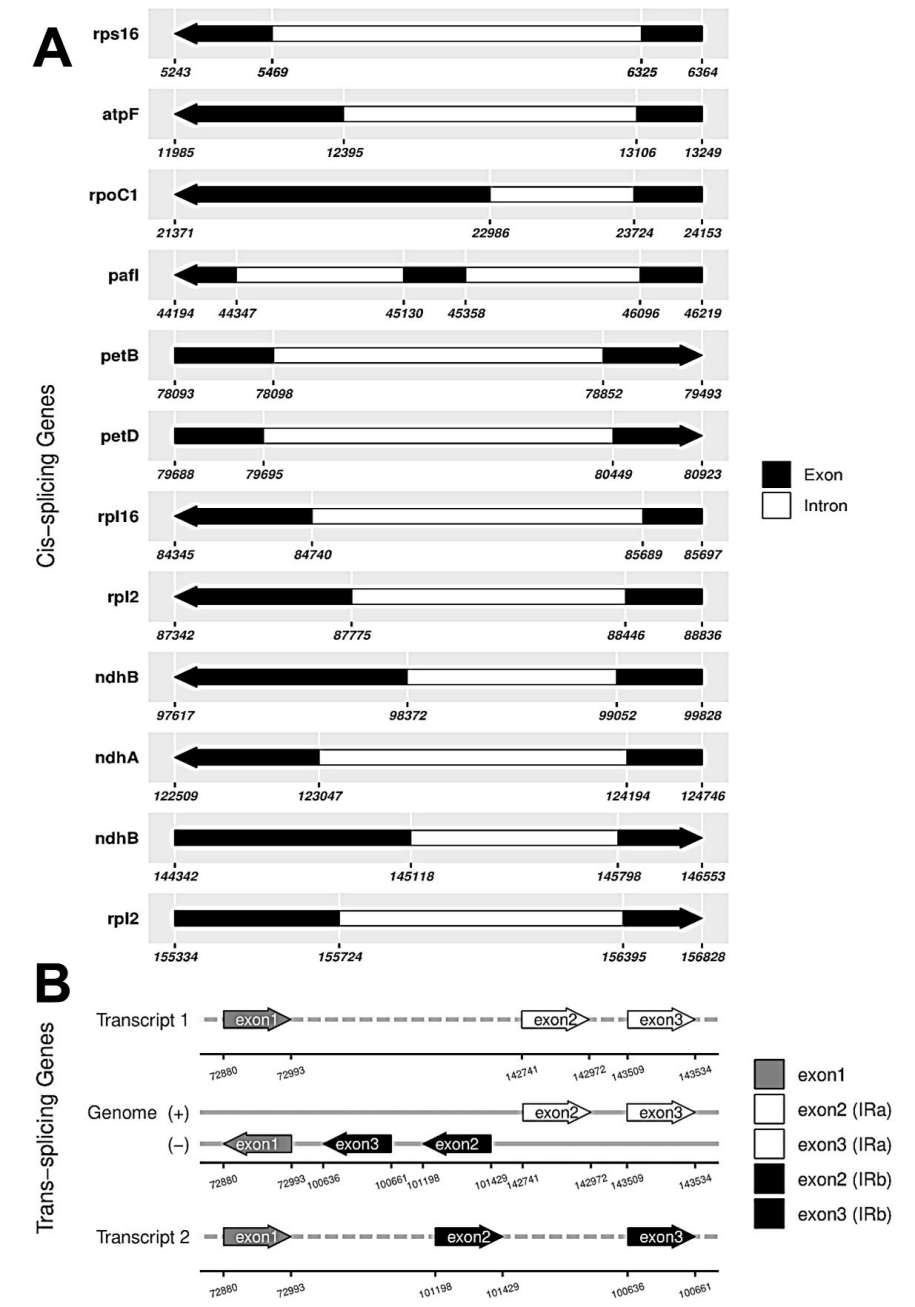
Structure of the genes that are difficult to annotate, including (A) cis-splicing genes, and (B) trans-splicing genes.

The minimum and average read mapping depths were 265× and 1038.5×, respectively (Fig. 2). The complete plastome sequence of *P. minima* (GenBank accession no. PP471919) was 156,973 bp in size. The genome has a typical quadripartite structure, consisting of a large single-copy (LSC), a small single-copy (SSC), and a pair of inverted repeats (IR_A_ and IR_B_) (Fig. 3), which were 87,196 bp, 18,447 bp and 25,665 bp in length, respectively. The total GC content was 37.5%. A total of 129 genes were annotated, including 84 CDSs, 37 tRNAs and eight rRNAs (Table 1). The annotation of the trans-splicing gene, rpl12, was identified, and for the cis-splicing genes, 16 contained one intron, i.e., *atp*F, *ndh*A, *ndh*B, *pet*B, *pet*D, *rpl*2, *rpl*16, *rpo*C1, *rps*16, *rrn*23, *trnA*-UGC, *trnG*-UCC, *trnI*-GAU, *trnK*-UUU, *trnL*-UAA and *trnV*-UAC; while two contained two introns, i.e., *paf*I, and *rps*12 (Fig. 4).

**FIGURE 5:**
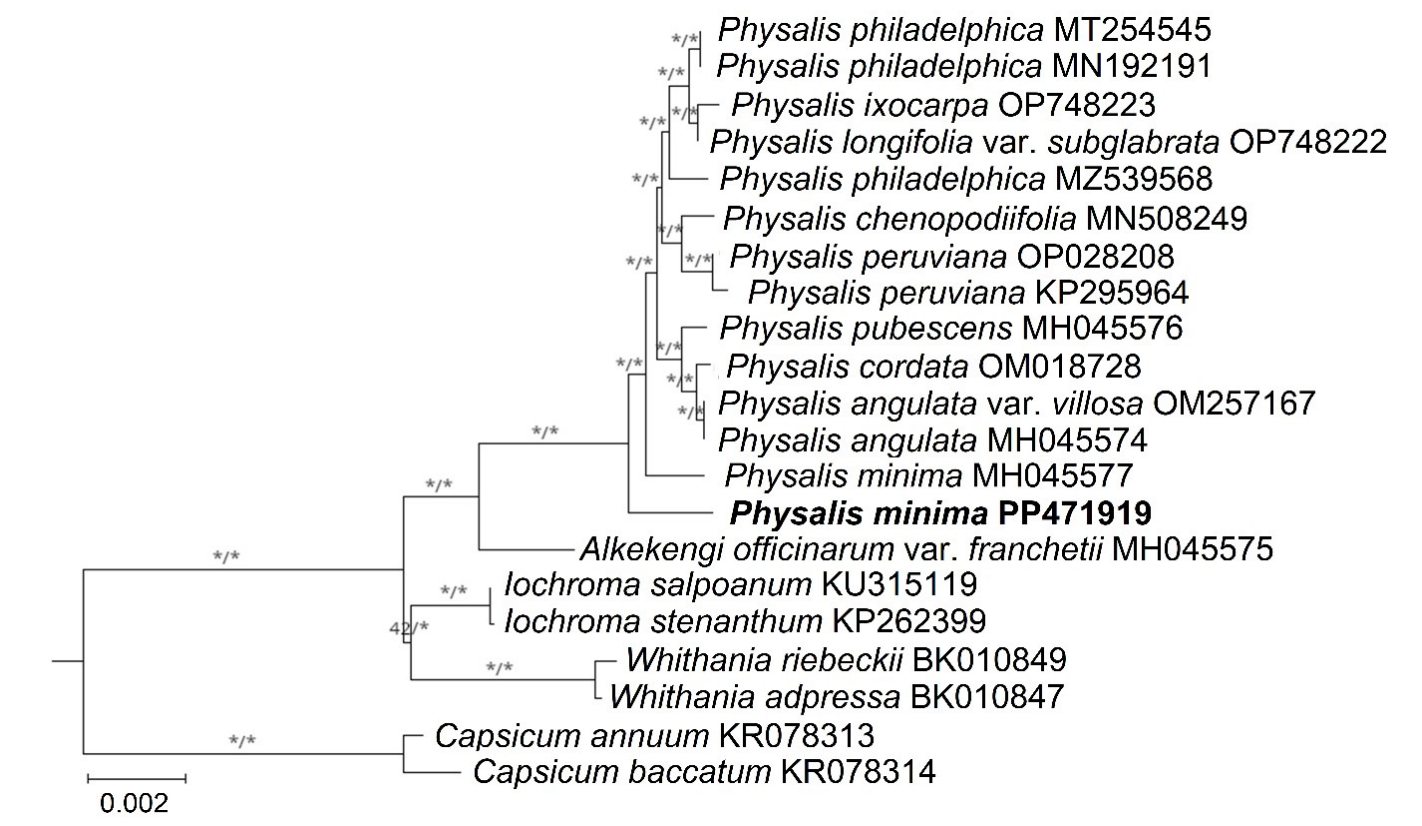
Phylogenetic analysis based on the complete plastid genome sequences of 19 Physaleae accessions using the maximum likelihood and Bayesian inference methods. Two closely related species, *Capsicum annuum* (GenBank accession no. KR078313), and *C. baccatum* (GenBank accession no. KR078314) were included as outgroups. Branch support values are indicated above each branch, in which a bootstrap support (left) value ≥ 75% and posterior probability (right) value ≥ 0.95, are considered strongly supported.

The alignment of the two *P. minima* complete plastome sequences was 157,330 bp in length. In the alignment, 458 polymorphic sites were identified, of which 259 were in the LSC region, 117 were in the SSC region and 82 were in the IR region. The intraspecific pairwise distance between the two P. minima accessions was 0.003. As both the ML and BI showed the same topology, only the ML tree was presented (Fig. 5). The phylogenetic relationship within Physalis was well-resolved, with bootstrap support (BS) values ≥ 75% and posterior probability (PP) values ≥ 0.95. However, a weak BS value was observed at the branch node that indicates the divergence between the Iochroma and Whithania clades (BS = 42%). Based on the current sampling size, both *P. minima* accessions did not cluster together under one clade; the *P. minima* from Malaysia was the first to diverge in the *Physalis* clade, followed by the accession from China.

## DISCUSSION

The complete plastome of *P. minima* shares key features with its congeneric species, i.e., *P. macrophysa* and *P. ixocarpa* (Zhang *et al*. 2022), reflecting a conserved plastome structure within *Physalis*. Pairwise comparisons with the accession from China revealed a close intraspecific relationship, with most variable sites in the LSC region and fewest in the IR region, similar to findings on *Ipomoea obscura* (Sudmoon *et al*. 2024). The low intraspecific pairwise distance observed between the two *P. minima* plastomes falls within the typical range of intraspecific plastome variation in angiosperms, which is reported to be generally less than 1.0% (Liu *et al*. 2024). This low divergence supports their conspecific status and reflects expected plastome-level variation within a species. Within the phylogenetic framework, it is expected that the limited sampling in this study may have reduced resolution at deeper nodes, while variations in analytical frameworks, including substitution models and bootstrap strategies, could significantly impact tree topology and support metrics. This can be explained through the discrepancies in branch support and the phylogenetic placement of *Iochroma* and *Withania*, as reported by Sandoval-Padilla *et al*. (2025). *Physalis minima* has recently been synonymised with *P. angulata* (POWO 2024); however, our phylogenetic analysis of complete plastome sequence suggests that they are two natural groups. In the ML tree, *P. angulata* is placed more closely with the American species, *P. cordata* and *P. pubescens*; while the two *P. minima* accessions form a separate lineage. The divergence between the Malaysian and Chinese accessions of *P. minima* may reflect geographic structuring and historical isolation, suggesting the presence of distinct evolutionary lineages. This observation has important implications for taxonomic reassessment, as well as conservation, since preserving regionally distinct lineages contributes to the maintenance of genetic diversity and species resilience. Additionally, such genomic divergence may correlate with differences in phytochemical profiles, reinforcing the importance of precise identification in ethnomedicinal contexts (Hao & Xiao 2015). From an ecological perspective, plastome variation may reflect local adaptation to environmental conditions, thereby offering insights for habitat-specific conservation planning (e.g. Hu *et al*. 2015). Given the unresolved taxonomy of *Physalis*, where combined plastid and nuclear markers have failed to fully resolve species boundaries (Pere *et al*. 2023; Whitson & Manos 2005), our results indicate that complete plastome sequences may better resolve these complex relationships. Notably, this study offers a foundation for future ecological and pharmaceutical research on *Physalis* by resolving taxonomic ambiguities and intraspecific variation, thereby enabling focuses investigations into medicinal properties, phytochemical diversity, and environmental adaptation to support sustainable utilisation and conservation. Nevertheless, the genomic data presented in this study could offer valuable insights for research on evolutionary patterns and genetic classification within Physalis and Solanaceae.

## CONCLUSION

This study reports the complete plastome of *Physalis minima* from Malaysia and provides the first comparative assessment with the previously published accession from China. The plastome structure was highly conserved, but genomic variation and phylogenetic analyses revealed clear genetic differentiation between the two accessions, supporting their recognition as distinct evolutionary lineages. These findings highlight the importance of complete plastome sequences in resolving taxonomic ambiguities within *Physalis* and provide valuable genomic resources for future studies on medicinal properties, phytochemical diversity and conservation of this ethnobotanically important species.

## Figures and Tables

**TABLE 1:** List of annotated genes in the plastid genome of *Physalis minima*, along with their group and function. Genes that are duplicated in the inverted repeat region are indicated with an asterisk (*).

Function	Gene group	Name of gene
Photosynthesis pathway	Photosystem I	*psa*A, *psa*B, *psa*C, *psa*I, *psa*J
	Photosystem II	*psb*A, *psb*B, *psb*C, *psb*D, *psb*E, *psb*F, *psb*H, *psb*I, *psb*J, *psb*K, *psb*L, *psb*M, *psb*T, *psb*Z
	ATP synthase	*atp*A, *atp*B, *atp*E, *atp*F, *atp*H, *atp*I
	NADH complex	*ndh*A, *ndh*B*, *ndh*C, *ndh*D, *ndh*E, *ndh*F, *ndh*G, *ndh*H, *ndh*I, *ndh*J, *ndh*K
	Cytochrome b6/f complex	*pet*A, *pet*B, *pet*D, *pet*G, *pet*L, *pet*N
	Large subunit of Rubisco	*rbc*L
	Photosystem biogenesis	*pbf*1
Structural RNAs	Transfer RNAs	*trnA*-UGC*, *trnC*-GCA, *trnD*-GUC, *trnE*-UUC, *trnF*-GAA, *trnfM*-CAU, *trnG*-GCC, *trnG*-UCC, *trnH*-GUG, *trnI*-CAU*, *trnI*-GAU*, *trnK*-UUU, *trnL*-CAA*, *trnL*-UAA, *trnL*-UAG, *trnM*-CAU, *trnN*-GUU*, *trnP*-UGG, *trnQ*-UUG, *trnR*-ACG*, *trnR*-UCU, *trnS*-GCU, *trnS*-GGA, *trnS*-UGA, *trnT*-GGU, *trnT*-UGU, *trnV*-GAC*, *trnV*-UAC, *trnW*-CCA, *trnY*-GUA
	Ribosomal RNAs	*rrn*4.5*, *rrn*5*, *rrn*16*, *rrn*23*
Genetic apparatus	Large subunit of ribosomal protein	*rpl*2*, *rpl1*4, *rpl*16, *rpl*20, *rpl*22, *rpl*23*, *rpl*32, *rpl*33, *rpl*36
	Small subunit of ribosomal protein	*rps*2, *rps*3, *rps*4, *rps*7*, *rps*8, *rps*11, *rps*12*, *rps*14, *rps*15, *rps*16, *rps*18, *rps*19
	Subunits of RNA polymerase	*paf*I, *paf*II, *rpo*A, *rpo*B, *rpo*C1, *rpo*C2
Others	Maturase	*mat*K
	Inner envelope membrane	*cem*A
	Cytochrome biogenesis protein	*ccs*A
	Fatty Acid synthesis	*acc*D
Unknown	Open-reading frame	*ycf*1*, *ycf*2*

## Data Availability

The genome sequence data that support the findings of this study are openly available in GenBank of NCBI at http://www.ncbi.nlm.nih.gov under the accession number PP471919. The associated BioProject, SRA, and BioSample numbers are PRJNA853926, SRR31280283 and SAMN44632072, respectively.
